# Novel locked nucleic acid (LNA)-based probe for the rapid identification of *Chlamydia suis* using real-time PCR

**DOI:** 10.1186/s12917-014-0225-4

**Published:** 2014-09-24

**Authors:** Paweł Lis, Aleksandra Kumala, Mirosław Spalinski, Krzysztof Rypula

**Affiliations:** Division of Infectious Diseases and Veterinary Administration, Department of Epizootiology with Clinic of Birds and Exotic Animals, Faculty of Veterinary Medicine, University of Environmental and Life Sciences, Grunwaldzki Square 45, 50-366 Wrocław, Poland; Veterinary Practice CHIRION, Błonie 3, 17-120 Brańsk

**Keywords:** Chlamydia suis, Pigs, Diagnostics, Real-time PCR, Probe, LNA

## Abstract

**Background:**

As the importance of chlamydial infections in pigs has become more obvious, a rapid and sensitive method to study the prevalence of *Chlamydia suis* in pig herds is required. Such a method should permit routine diagnostic tests for herds with clinical and subclinical infections, without the need for *Chlamydia* culture.

**Results:**

The main objective of this study was to develop a specific and rapid method for detecting *C. suis* in swine herds. A real-time PCR assay using a single locked nucleic acid (LNA)-containing probe specific for *C. suis* was developed based on the previously described 28S rDNA fragment used to identify Chlamydiales. Use of LNA nucleotides enabled the single probe to target a short, specific fragment of the 23S rRNA. The probe showed high specificity for *C. suis* and did not show any cross-reactivity with other *Chlamydia* or *Chlamydophila* species nor with swine DNA. All of the 86 tested field isolates, earlier identified as *C. suis*, were confirmed as positive using the newly developed assay.

**Conclusions:**

Using single LNA-based *C. suis-*specific probe allowed rapid and simple identification of this pathogen without requiring sequencing analysis and culturing. The proposed method may be used to study the prevalence of *C. suis* infection in pig herds and as a routine diagnostic test for herds with clinical and subclinical infection.

## Background

*Chlamydia suis*, *Chlamydophila abortus*, *Chlamydophila psittaci* and *Chlamydophila pecorum* infections have been identified in pigs. Recent studies indicate that these infections occur more frequently than originally thought [1-4]. In particular, the study ‘Animal Chlamydiosis and its zoonotic implications’ by Action 855 of the European Cooperation in Science and Technology brought the economic importance of chlamydial infections in pigs into sharp focus. Chlamydial infections have been implicated in a wide spectrum of clinical disorders, including reproductive disorders, ocular and respiratory problems (e.g. conjunctivitis and pneumonia), enteritis, pleuritis, and polyarthritis [4]. Chlamydia can cause both acute and latent disease forms, and very often, chlamydial diseases are asymptomatic.

*C. suis* appears to be both common and widespread, often occurring in mixed infections with *Cp. abortus*, *Cp. psittaci*, and *Cp. pecorum* [5]. The pig intestine is considered to be the natural reservoir for *C. suis* [6]. The latent presence of chlamydia in the alimentary tract can result in systemic infection when the bacteria reach the epithelial and cellular layers. When the lymph system is affected, the process is called chlamydiosis [7]. *C. suis* has been identified with high prevalence in growing pigs with or without diarrhoea, as well as in finisher pigs with or without conjunctivitis [4]. A correlation has been noted between samples with a high degree of infection and the presence of clinical signs of chlamydial disease. The use of intensive pig production systems might increase the risk of chlamydial disease in pigs [6].

In gnotobiotic pig challenge studies, *C. suis* caused diarrhoea in young piglets, and chlamydial antigens were identified in these animals. Chlamydia was also found in enterocytes isolated from the intestinal tissue and faeces [7,8]. Experimental aerosol challenge of 7-day-old pigs with *C. suis* confirmed the pathogenic potential of *C. suis* in the porcine respiratory system. All infected animals showed clinical signs, including dry cough, serous nasal discharge, and severe dyspnoea with wheezing, shortness of breath, and breathlessness. The body temperatures of the infected animals rose above 40°C [9,10]. Another study conducted in Germany and Switzerland demonstrated a high prevalence of *C. suis* in the eyes of pigs with ocular symptoms [11]. *C. suis* has been investigated in pigs in association with reproductive disorders, such as return to oestrus (with early embryonic death in >50% of sows) and inferior semen quality (with decreased sperm cell motility and death of >50% of sperm cells). These pathologies have been demonstrated on farms in Belgium, Cyprus, Estonia, Germany, Israel, and Switzerland [1,12-14]. *C. suis* alone may be responsible for diarrhoea in young piglets, resulting in the partial loss of litters and increased owner costs incurred on veterinary care. However, the clinical importance of these disorders appears to decrease in concert with the growth of the animals [8]. Necropsy and pathological investigations may be a first step in the analysis of the source of losses. In a study of field infections, however, pathogen (*C. suis*)-related lesions appeared in 50% of the samples examined [4].

Using a locked nucleic acid (LNA)-containing probe, in this study, we evaluated a real-time PCR assay specific for *C. suis*. This assay is based on the previously described 28S rDNA that was used for the identification of Chlamydiales [15]. The LNA nucleotides, which have an additional methylene bridge between the 2′ oxygen and the 4′ carbon of the ribose ring, exhibit much higher binding affinity than unmodified nucleotides. Compared to standard DNA probes, the locked structure results in higher sensitivity and increased specificity of the LNA-containing probes [16]. These characteristics enable the use of much shorter probes (7–10 nucleotides) and simplify the identification of appropriate regions for probe hybridization.

## Methods

### Sample collection

This study included 86 field archival isolates of *C. suis* from years 2011–2013, which were derived from clinically healthy animals and pigs with clinical disorders (i.e. conjunctivitis cases and reproductive disorders in sows). The research was conducted in closed-loop farms with 20–120 adult sows in each basic herd. Ten farms were tested. The required sample size was computed in WinEpiscope 2.0 (EPIDECON), assuming an expected prevalence of 30% and a confidence level of 95%.

Conjunctival swabs from both eyes and a swab from the vaginal vestibule were collected from each sow. The specimens were stabilized by using the PAXgene Tissue Stabilizer (Qiagen, Poland) and stored at −80°C. Reference strains of positive DNA for other chlamydial organisms were as follows: *C. suis* VR-1474 (ATCC, USA), *Chlamydia trachomatis* and *Chlamydophila abortus* (NRVI, Puławy, Poland), *Chlamydophila felis* strain 905 (Merial, France), and *Chlamydophila psittaci* (field isolate obtained from birds, shared by Dr. T. Piasecki, Wroclaw University of Environmental and Life Sciences, Poland). *Mycoplasma* spp. ATCC 2391 (LGC Standards, Poland) and *S. epidermidis* ATCC 35984 (PAN, Wroclaw, Poland) were included as controls.

DNA was extracted and purified with a GeneJET Viral DNA and RNA Purification Kit, according to the manufacturer’s manual (Thermo Scientific, Lithuania). All DNA samples were tested by using the qPCR procedure described by Everett et al. [15] and sequencing the obtained products.

### Design of the assay

**Figure 1:**
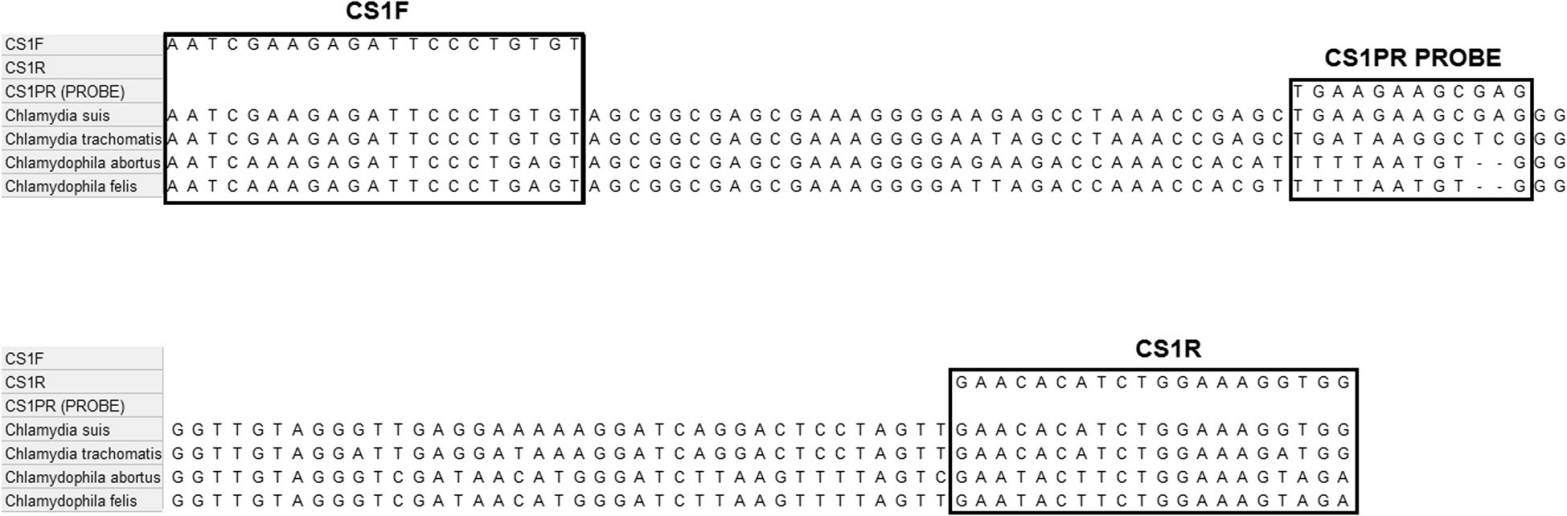
**Sequence recognized by the designed** ***C. suis*** **specific LNA probe and primers, taking into account analogous sequences in the closely related** ***Chlamydiaceae*** **(** ***C. trachomatis*** **,** ***Cp. abortus*** **,** ***Cp. felis*** **).**

Primers and probe were designed by using the chlamydial sequences available in GenBank and were evaluated with Primer3 [17]. Table 1 shows the list of sequences of the 23S rRNA gene and the genome sequences from GenBank used in the development of the primers and probes. Sequences were aligned and analysed using MEGA6 software [18]. Figure 1 shows the sequence recognized by the *C. suis*-specific LNA probe and the homologous sequences from the closely related *C. trachomatis* and *Cp. abortus* genomes.

**Table 1:** **Sequences from GenBank used in the development of primers and probe**

**Species**	**Strain**	**NCBI accession number**
*Chlamydia muridarum*	Nigg CM972	CP006975.1
*Chlamydia suis*	R19	AF481047.1
	R24	U68428.1
	R27	U68429.1
	MS04	DQ118376.1
	H5	U68427.1
*Chlamydia trachomatis*	C/TW-3	CP006945.1
*Chlamydophila abortus*	S26/3	CR848038.1
*Chlamydophila caviae*	GPIC	AE015925.1
*Chlamydophila felis*	Fe/C-56	NR_076260.1
*Chlamydophila pecorum*	P787	CP004035.1
*Chlamydophila pneumoniae*	CWL029	NR_076161.1
*Chlamydophila psittaci*	WS/RT/E30	CP003794.1

Products obtained with real-time PCR were sequenced (Genomed, Poland) and identified using BLAST (blast.ncbi.nlm.nih.gov). Oligonucleotides were synthesized by Sigma-Aldrich. Primer and probe sequences used for verification of the diagnostic method are provided in Table 2.

**Table 2:** **Sequences of primers and probes used during the study**

	**Sequence (5′-3′)**	**Source**
CS1F	AATCGAAGAGATTCCCTGTGT	This study
CS1R	CCACCTTTCCAGATGTGTTC	This study
CS1PR	FAM– T [+G] A [+A] [+G] A [+A] GC [+G] [+A] G –BHQ1	This study
TQF	GAAAAGAACCCTTGTTAAGGGAG	Everett et al. [15]
TQR	CTTAACTCCCTGGCTCATCATG	Everett et al. [15]
Probe	FAM-CAAAAGGCACGCCGTCAAC-TAMRA	Everett et al. [15]

[+N] – LNA BASES.

To validate each new set of primers, a classic PCR was performed, followed by qPCR with SYBR Green. Next, the qPCR was tested with the designed LNA probe. The primer sets proposed in this study were specific for the 23S rRNA gene. Each sample was tested in triplicate. Serial 10-fold dilutions were performed on the *C. suis*-positive sample to assess the test sensitivity. The spectrophotometrically determined mean DNA concentration of the reference strain was 6 ng/μl.

### PCR procedures

The classic PCR was performed using 2.5 μl of DreamTaq Green buffer, 0.5 μl of dNTPs (10 mM), 1 μl of CS1F (10 μM), 1 μl of CS1R (10 μM), 0.2 μl of DreamTaq DNA polymerase (Thermo Scientific), and 18.8 μl of water. Each reaction included 1 μl of matrix DNA. The cycling conditions were 3:00 at 95°C; 40 cycles of 95°C for 0:30, 48°C for 0:30, 72°C for 0:30; and 5:00 at 72°C.

Reaction mixtures for real-time PCR with the TQF/TQR primers and probe, as described by Everett et al. [15], contained 10 μl of KAPA PROBE FAST Bio-Rad iCycler 2× qPCR Master Mix (KapaBiosystems, USA), 0.3 μl of each primer (10 μM), 0.2 μl of the probe (10 μM), 4.2 μl of miliQ water, and 5 μl of genomic DNA. The real-time PCR cycling included 40 cycles of 0:15 at 95°C and 1:00 at 60.5°C, with an initial denaturation for 3:00 at 94°C. Real-time PCR was performed in a Bio-Rad iQ5 (Bio-Rad, Poland). The following reaction mixture was used for the real-time PCR with the CS1PR probe: 10 μl of KAPA PROBE FAST Bio-Rad iCycler 2× qPCR Master Mix, 0.4 μl of each primer (10 μM), 0.4 μl of probe (10 μM), 7.8 μl of miliQ water, and 1 μl of gDNA. The cycling conditions were 40 cycles of 0:03 at 95°C and 0:20 at 48°C, with an initial denaturation of 3 min at 94°C. Real-time PCR was performed using a Bio-Rad iQ5. The SYBR Green assay was performed using the same parameters and the KAPA SYBR® FAST Bio-Rad iCycler 2× qPCR Master Mix.

### Ethic statement

The Ethical Committee for Animal Experiments, Wrocław, Poland, approved this study and all owners provided informed consent prior to initiation.

## Results

**Figure 2:**
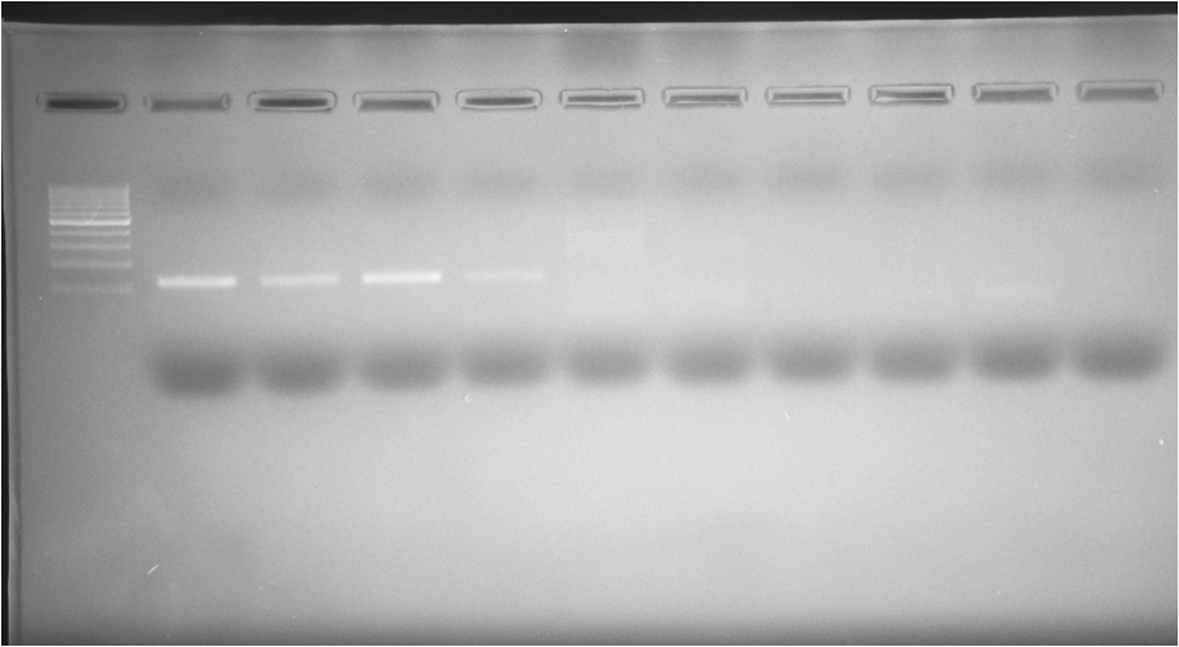
**Results of the amplification of the fragment of 23S rRNA gene using CS1F/CS1R primers.** On the gel, from the left: 100 bp ladder; *C. suis*; *Cp. abortus*; *C. trachomatis*; *Cp. felis*; *Cp. psittaci*; *M. felis*; *S.epidermidis*; swine DNA; NTC.

Traditional PCR reactions were conducted to test the specificity of the designed primers. The CS1F and CS1R primers amplified a 128-bp fragment of the 23S rRNA gene, showing a positive signal in PCRs using *C. suis*, *Cp. abortus*, *C. trachomatis,* and *Cp. felis* DNA. No products were observed in reactions containing *Cp. psittaci*, *M. felis*, *S. epidermidis,* or swine DNA (Figure 2).

**Figure 3:**
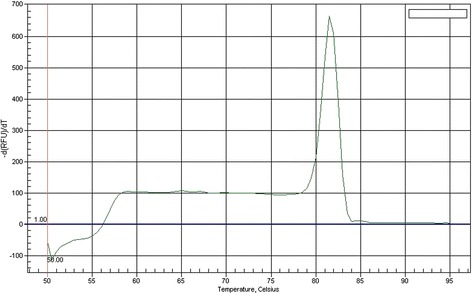
**Melting curve of the product of the amplification of the** ***C. suis*** **23S rRNA gene fragment using CS1F/CS1R primers.**

Subsequently, real-time PCR was performed using the CS1F and CS1R primers and *C. suis* gDNA. One clear peak was visible in the melting curve analysis, indicating that a single amplicon was obtained in the real-time PCR procedure (Figure 3).

The PCR product was sequenced, and the identified sequence was identical to the *C. suis* R19 23S rRNA gene fragment sequence obtained from GenBank (AF481047.1).

The CS1PR probe was designed to recognize *C. suis* specifically, while excluding other chlamydial sequences available in GenBank to avoid any cross-reactivity. Use of LNA nucleotides enabled us to develop a short (12 nt), yet specific, probe. Real-time PCR using the CS1PR probe showed no positive signals when DNA isolated from *C. trachomatis*, *Cp. abortus*, *Cp. psittaci*, *Cp. felis*, *M. felis*, *S. epidermidis*, or swine DNA was used as a template.

**Figure 4:**
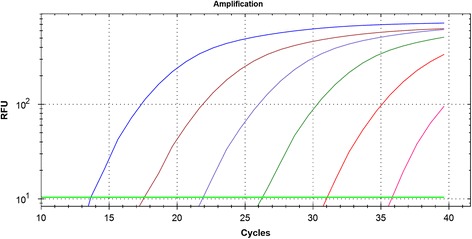
**Real time-PCR assay with CS1PR probe performed on serial 10-fold dilutions of** ***C. suis*** **genomic DNA (6 ng to 60 fg of DNA).**

Six serial 10-fold dilutions of *C. suis* gDNA isolated from pure culture were used to determine the assay sensitivity. The range of detection of the assay was between a C_T_ of 13 (6 ng of genomic DNA) and a C_T_ of 36 (60 fg of genomic DNA), indicating that the lowest amount of genomic DNA detectable by the assay was 60 fg (about 50 copies; Figure 4). Correlation coefficients of the standard curves were all above 0.96, and the efficiency of the reaction was 98.9% (slope: −3.349 ± 0.3727). All 86 of the field isolates selected with the *Chlamydiaceae*-specific probe and identified as *C. suis* by sequencing of the PCR product as in Everett et al. [15] were confirmed as positive by the CS1PR probe.

## Discussion

The rate of *Chlamydiaceae* seroprevalence in pig farms across European countries, including Germany, Belgium, and Switzerland, differs greatly. Frequently, seroprevalence depends on the age of pigs examined, although seropositivity has been confirmed in all phases of pig production. The lowest rate (6.9%) was observed in piglets younger than 4 weeks old, whereas the rate reached 48.1% in older pigs [1]. In sows specifically, the reported seroprevalence ranges from 33% to 72% [12]. In Belgian fattening farms, sow seropositivity has been reported to be as high as 96.5% [2]. A previous study reported seropositivity to *C. suis* in pigs from all 11 farms investigated, with seropositivity rates ranging from 20% to 100% [19].

Guscetti et al. [20] investigated *C. suis* infection in gnotobiotic piglets. Animals were inoculated with egg-grown chlamydiae at 2–3 days of age and were observed for clinical signs. Animals were sacrificed and necropsied at 2–13 days post-inoculation, and serological tests were performed (complement fixation test and ELISA). Clinical symptoms, including diarrhoea, slight and transient anorexia, weakness, and body weight loss, were observed. Immunohistochemistry and ELISA revealed that chlamydial replication was particularly marked at 2–4 post-inoculation and primarily located in the small intestine. However, all sera were negative for antichlamydial antibodies [20]. In general, intensively kept pigs are predisposed to ocular chlamydial infection associated with conjunctivitis [11].

Cell culture is widely regarded as the gold standard in chlamydial diagnosis. However, this method has numerous limitations, particularly in the case of *C. suis*, which displays limited growth in commercial cell lines [21]. In commercial laboratories, the species-specific identification of pathogens is uncommon because specimens collected in the field are generally diagnosed only to the *Chlamydiaceae* family level. Consequently, the epidemiological situation remains largely unknown, as individual pathogens are not considered. As a diagnostic approach, PCR detection of *C. suis* provides more satisfactory results than direct culture, particularly if percent positivity is considered (42% vs. 33%) [22]. The sensitivity and specificity of the reaction proposed by Sachse et al. were 94.4% and 81.0%, respectively [22].

The very high sensitivity of real-time PCR enables direct identification from swabs without prior propagation of the pathogen by cell culture. Current PCR tests proposed by various researchers for the identification of *Chlamydiaceae* in pigs target the ompA, 16S-23S rRNA, or incA gene. Species-specific real-time PCR tests proposed for pigs detect the ribosomal intergenic spacer and domain I of the 23S rRNA gene [6]. Using the PCR-RFLP method described by Demkin and Zimin [23], we were unable to obtain product using material isolated from swabs, indicating that classical PCR is was not sensitive enough for identification of *Chlamydiaceae* from this type of material without culturing (unpublished data).

In this study, we designed a real-time PCR assay for the identification of *C. suis*. To our knowledge, this is the first report of a *C. suis*-specific real-time PCR method that allows the highly specific and sensitive detection and species-level identification of the pathogen with a single oligonucleotide LNA-based probe and without requiring sequencing analysis. Using LNA nucleotides allowed us to target a short, specific fragment of the 23S rRNA gene for development of the *C. suis-*specific probe. The proposed method may be used to study the prevalence of *C. suis* infection in pig herds and as a routine diagnostic test for herds with clinical and subclinical infection. Species-specific identification may be important therapeutically, because the only known stable tetracycline-resistant strains of *Chlamydia* are *C. suis*.

## Conclusions

We designed a single LNA-based *C. suis-*specific probe that allowed rapid and simple identification of this pathogen using real-time PCR, without requiring sequencing analysis and culturing. 86 *C. suis*-positive samples were tested and successfully identified using the newly designed probe, showing high specificity and sensitivity of the described method. The proposed assay may be used to study the prevalence of *C. suis* infection and as a routine diagnostic test for herds with clinical and subclinical infection.
